# Developmental diversity in meristem organization: lessons from non-canonical plant body plans for biotechnology

**DOI:** 10.3389/fpls.2026.1654676

**Published:** 2026-07-06

**Authors:** Carlo Massimo Pozzi, Alberto Spada

**Affiliations:** Department of Agricultural and Environmental Sciences, University of Milano “La Statale”, Milan, Italy

**Keywords:** *bauplan*, biotechnological prospectives, developmental differences, meristem, non-canonical plant body

## Abstract

The canonical plant *bauplan* is governed by the activity of the shoot apical meristem (SAM), a structural hub established during embryogenesis that produces repeating modular units known as phytomers. However, extreme morphological divergence in families such as Podostemaceae, Lemnaceae, and Gesneriaceae reveals the plasticity of plant developmental programs. In several lineages, the canonical deployment of meristematic programs is modified, displaced, or developmentally reduced, giving rise to divergent but evolutionarily successful body plans. This review explores the molecular interplay of meristematic regulators, characterizes the growth habits and genomic landscapes of these non-canonical lineages, and discusses how they may inform experimentally testable frameworks for engineering next-generation crop architectures.

## Introduction

### Structure of the canonical SAM and molecular interplay

Established during embryogenesis, the SAM drives primary growth by maintaining a pool of undifferentiated stem cells while initiating lateral organs. This activity generates repeating phytomers, modules consisting of an internode, a node with a leaf, and an axillary meristem (AM), which collectively form the aerial part of the plant. The SAM and the floral meristem determine many agronomically important traits such as fruit size and number, phyllotaxis, and stem architecture (Lindsay et al., 2024). The structure of the SAM features a Central Zone (CZ), with stem cells; a Peripheral Zone, which generates lateral organs, and AM that are formed at the boundary between developing leaves and SAM; an Organizing Center (OC), i.e. few cells just below the CZ, which regulates cell fate and SAM patterning. Deep within the meristem, the rib zone is responsible for the proliferation of internal stem tissues (Kuznetsova et al., 2023). Molecular regulation of SAM appears to be conserved across species (Agarwal et al., 2022). The key gene players are *CLAVATA (CLV)* and *WUSCHEL (WUS)*. WUS is a homeobox transcription factor expressed in the OC. WUS protein moves through plasmodesmata into the overlying CZ, where it specifies stem cell identity and induces the expression of the signaling peptide CLAVATA3 (CLV3). In turn, CLV3 acts as a ligand for CLV1, CLV2 and CORYNE (CRN) receptors in the CZ (Lin and Irish, 2025). Upon perception, this signaling cascade moves back into the OC to inhibit *WUS* transcription. This feedback ensures a constant pool of stem cells.

Essential to this process is the collaboration with *SHOOT MERISTEM LESS* (*STM*) and *CUP SHAPED COTYLEDONS* (*CUC*). *STM* is a class-1 *KNOX* gene, expressed in the SAM, but not in lateral organ primordia, and it maintains cells in an undifferentiated state. *CUC* belongs to the *NAC* family and is required for the activation of *STM* during embryogenesis. In turn, *STM* controls *CUC* expression post-embryonically at organ boundary domains, excluding it from the SAM’s center to maintain the apical dome (Spinelli et al., 2011). The MYB-domain genes *ASYMMETRIC LEAVES1/ROUGHSHEATH2* (*ARP*) promote cell differentiation and specify leaf polarity. In an antagonistic relationship, ARP represses *STM* in leaf primordia to allow for differentiation, while *STM* prevents *ARP* expression within the meristem to preserve the niche (Eshed Williams, 2021; Lv et al., 2023). The regulatory network governing stem cell maintenance in the SAM appears less well-defined in monocots than in dicots, although both groups utilize the *CLV*/*WUS* negative feedback loop pathway (Dresselhaus et al., 2025). While the maintenance of the primary stem cell pool establishes the developmental foundation, mature plant architecture is ultimately driven by shoot branching—a conserved process that generates complex patterns through the iterative, context-dependent regulation of axillary meristems (Chachar et al., 2025). Major regulators of branching include *TILLER ANGLE CONTROL 1* (*TAC1*) in rice, and *BRANCHED1* (*BRC1*) in Arabidopsis (Hollender et al., 2020), while additional components involved in tillering in different species include *MONOCULM 1* (*MOC1*), *IDEAL PLANT ARCHITECTURE 1* (*IPA1*), and *MORE AXILLARY GROWTH* (*MAX*) (Chachar et al., 2025). Leaf complexity and vascular patterning are governed by interconnected regulatory networks involving *ASYMMETRIC LEAVES 1* (*AS1*), *TEOSINTE BRANCHED1/CYCLOIDEA/PROLIFERATING CELL FACTOR* (*TCP*), *ANGUSTIFOLIA3* (*AN3*), and *NARROW LEAF1* (*NAL1*) (You et al., 2022). The precise activity of these factors is modulated by post-translational mechanisms that restrict tissue overgrowth. For example, the ubiquitin-activated peptidase DA1 negatively regulates leaf size by cleaving targets such as maize *TCP*, and rice *PCF1*, *PCF2*, whereas *CUC* genes positively regulate shoot apical and axillary meristem formation. ANGUSTIFOLIA3/GIF1, a *GROWTH REGULATING FACTOR* (GRF), promotes leaf growth by stimulating cell division, and NAL1 influences the spatial distribution of vascular tissues (Guo et al., 2022). In addition, across various plant species, the indeterminate growth of lateral organs is regulated by the *Auxin-Regulated Gene involved in Organ Size* (*ARGOS*), which promotes leaf growth by enhancing *AINTEGUMENTA* (*ANT*) and D-type cyclins *CYCD3;1* (Schneider et al., 2024).

## Discussion

### Non-canonical plant body plans

Plant lineages in which the distinctions and origins of organs have become blurred represent successful evolutionary outcomes rather than developmental anomalies. The dicots Podostemaceae and Gesneriaceae, and monocots such as Lemnaceae, illustrate how changes in the spatial deployment, maintenance, or reduction of conserved developmental programs can generate non-canonical body plans from the canonical plant *bauplan* (Pozzi et al., 2024). Here, these taxa are discussed as comparative developmental systems, not as deviations from an idealized model.

**Figure 1:**
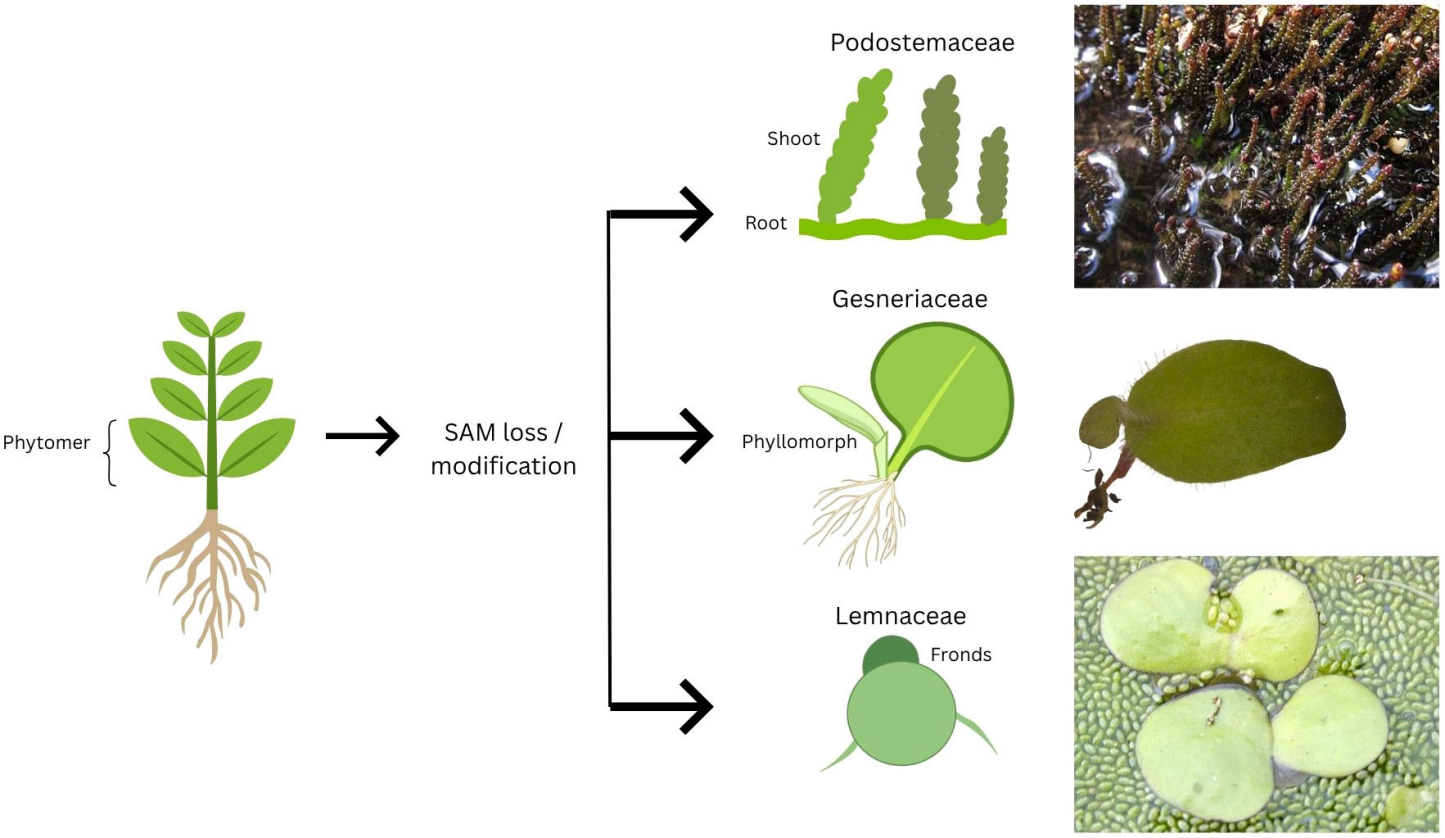
Evolutionary diversification of plant body plans through SAM modification. Schematic representation of the transition from canonical, phytomer-based architecture (left) to highly derived morphologies observed in selected non-canonical lineages (right). Divergence in the deployment, maintenance, or reduction of the Shoot Apical Meristem (SAM) program is associated with the evolution of alternative structural units, including the root-borne shoots of Podostemaceae, the phyllomorphs of some Gesneriaceae, and the miniaturized vegetative fronds of Lemnaceae. The figure is intended as a simplified guide for readers unfamiliar with these taxa and should be interpreted together with the morphological descriptions in the text. Images of Podostemaceae (*Cladopus queenslandicus*) and Lemnaceae (*Wolffia globosa* and, with larger fronds, *Spirodela polyrhiza*) are adapted from Sworboys, https://www.inaturalist.org/photos/110891707, Cc-by-4.0, and from Elkin Meriño, https://www.inaturalist.org/photos/345919967, CC BY, respectively. The Gesneriaceae member (image by the authors) is *Streptocarpus rexii*.

The Gesneriaceae (**Figure 1**) include species with a conventional architecture and a functional SAM, as well as members of the subfamily Didymocarpoideae (e.g., *Streptocarpus*) in which the phytomer is replaced by a phyllomorph, a composite structure combining leaf and stem characteristics that develops in the absence of a functional SAM from embryogenesis onward. During germination, two isocotyledons are formed. Subsequently, anisocotyly occurs, whereby one cotyledon (macrocotyledon) continues to grow and is sustained by three non-canonical meristems: the basal meristem (Bm), responsible for lamina expansion; the petiolode meristem (Pm), which drives midrib/petiole elongation; and the groove meristem (Gm), which initiates new organs, namely phyllomorphs, that combine features of both a leaf and a stem (Jong and Burtt, 1975; Mantegazza et al., 2009). Additional phyllomorphs or inflorescences are produced from the Gm of pre-existing phyllomorphs. Anisocotyl and phyllomorph formation enable rapid addition of photosynthetic tissue and are associated with survival under conditions of limited light availability (Nishii et al., 2017). Some Gesneriaceae therefore provide a compelling case of altered meristem deployment, originating from an evolutionary trend within the Lamiales lineage. As demonstrated by Nishii et al. (2017), this group exhibits a progressive expansion of *STM* expression and cell division activity from the SAM into cotyledons and leaf primordia. In Streptocarpus, this shift is so pronounced that no SAM forms during embryogenesis; instead, pluripotency is maintained via the heterotopic expression of *STM* and *WUS* within the macrocotyledon’s meristematic regions (Bm, Pm, and Gm). The resulting co-expression of *STM* and *ARP* (Nishii et al., 2010) mirrors the regulation seen in compound-leaved plants like *Solanum lycopersicum*, supporting the hypothesis that phyllomorphs function as indeterminate leaf-stem hybrids driven by spatially extended meristematic programs. These molecular insights are supported by increasing genomic resources for *Streptocarpus rexii*, including a transcriptome and SNP-based genetic maps (Chiara et al., 2013; Chen et al., 2018). Nevertheless, functional mutant evidence remains limited, and the underlying networks may follow general principles of meristem formation while using different spatial contexts or regulatory players.

The Podostemaceae (**Figure 1**) display pronounced morphological ambiguity, with a partial blurring of the distinction between root and shoot that is associated with life in fast-flowing tropical rivers. As aquatic haptophytes, their body plan is reduced to a dorsiventrally flattened, photosynthetic thallus derived from roots, enabling colonization of submerged rock surfaces in fast-flowing water and reducing water resistance. In the subfamily Podostemoideae (e.g., *Hydrobryum*), the embryonic SAM is absent, and the plant body consists of an adventitious root arising from the lateral side of the hypocotyl. This root progressively forms adventitious shoots. Adventitious shoots repeatedly undergo a process of leaf formation in which each new leaf arises at the base of an existing leaf (Sehgal et al., 2002; Koi and Kato, 2010). In the subfamily Podostemoideae, orthologs of *STM*, *WUS*, *ARP*, and *CUC* have been characterized, and their expression patterns largely correspond to those observed in model plants (Fujinami and Imaichi, 2015). However, none of these genes is expressed during embryogenesis. Adventitious roots arise from the hypocotyl, and the WUS ortholog is expressed at the site near the root apical meristem (RAM) where the shoot develops from the root (Mohan Ram and Sehgal, 1997; Suzuki et al., 2002; Kita and Kato, 2005). When the shoot arises, STM is expressed in the leaf primordia, and later *ARP* becomes localized distally from *STM*, at the distal part of the leaves. The origin of adventitious shoots near the RAM may be due to ectopic *WUS* expression, as observed in Arabidopsis (Rashid et al., 2007; Katayama et al., 2010). In newly developing shoots, *CUC3* and *STM* are expressed in an almost mutually exclusive manner (Katayama et al., 2019). Crucially, the molecular dissection of this family is now feasible, supported by the availability of both a reference genome and comprehensive transcriptomic datasets for *Cladopus chinensis* (Podostemoideae) (Xue et al., 2020). In this species, evidence of ancient whole-genome duplications, subsequent gene loss, and elevated mutation rates has been interpreted in relation to evolution in harsh, high-UV environments (Katayama et al., 2022). Such duplication, fractionation, gene loss, and regulatory-network remodeling are well-established features of plant genome evolution and are relevant here as a possible context for developmental diversification rather than as exceptional processes (De Smet and Van de Peer, 2012; Soltis et al., 2015). Members of gene families involved in shoot development have been annotated, but their expression patterns and signaling pathways remain unstudied.

The Lemnaceae (duckweeds) (**Figure 1**) are a family of aquatic monocots characterized by extreme morphological reduction coupled with rapid growth. Species in this family lack a SAM and a typical phytomeric structure in both the embryo and the adult plant, and instead develop a frond, a modified leaf, or a highly condensed shoot system (Lemon and Posluszny, 2000). During embryogenesis, the first frond originates from the proembryo, which arises from one or two cells located below the epidermis (Maheshwari and Kapil, 1963). Subsequently, a ‘daughter frond’ emerges from meristematic-like cells within the developing tissue and eventually detaches. This process – in the absence of the SAM - initiates a clonal cascade, where new fronds successively arise from the base of the preceding daughter frond (Lemon and Posluszny, 2000; Yang et al., 2021). Members of this species reproduce mainly by budding and double their biomass every 48–72 hours, thus quickly colonizing aquatic surfaces under favorable conditions (Zhang et al., 2020).

Despite the availability of several sequenced genomes (Michael et al., 2017; Hoang et al., 2020; Michael et al., 2020; Abramson et al., 2022; Ernst et al., 2025), the spatiotemporal expression of meristematic regulators in the Lemnaceae remains largely uncharacterized. Comparative genomics within this family suggests that, in some lineages, morphological simplification can coincide with substantial genomic variation or genome expansion. However, this relationship should be interpreted cautiously because genome size evolution is context-dependent and may be constrained by ecological factors, including nitrogen and phosphorus availability.

Taken together, Gesneriaceae, Podostemaceae, and Lemnaceae show that divergent plant architectures can arise through the altered spatial deployment, reduction, or reorganization of conserved developmental programs. However, the molecular interpretation of these systems remains incomplete. In most cases, the expression domains, regulatory interactions, and functional roles of candidate genes such as *WOX/WUS*, *ARP/AS1*-like factors, *TCP*-related regulators, *CUC*, and components of hormone-dependent feedback or feedforward networks have not yet been fully characterized in these lineages. These genes should therefore be regarded as plausible entry points for investigation rather than as validated determinants of the observed body plans.

### Biotechnological perspectives from non-canonical development

Non-canonical plant body plans offer useful comparative systems for exploring how conserved developmental programs can be redeployed, reduced, or reorganized under specific ecological conditions. Gesneriaceae, Podostemaceae, and Lemnaceae may help identify developmental principles and candidate regulatory modules whose relevance for agronomically useful phenotypes could be experimentally tested in crop systems.

Enhanced vegetative propagation and regeneration are agronomically relevant traits, particularly in crops constrained by transformation or regeneration bottlenecks, such as cereals, legumes, woody crops, and industrial species. Gesneriaceae offer comparative examples of organ formation from non-canonical meristematic regions, suggesting that leaf-associated tissues may retain or re-acquire organogenic competence. These systems may help identify regulatory principles for direct organogenesis from somatic tissues and improve transformation pipelines in recalcitrant species (Yang et al., 2024). Candidate modules include *WUS/WOX*-related regulators, *AS1/ARP*-like factors, *TCP*-associated growth regulators, and chromatin states permissive to morphogenic programs. Recent advances using *WUS2*, *BABYBOOM* (*BBM*) and *TaWOX*-related approaches show that morphogenic competence can be experimentally manipulated in crops (Maher et al., 2020; Basu and Parida, 2023; Jiang et al., 2024). These studies support the broader rationale for exploring non-canonical developmental systems as sources of testable regulatory hypotheses for crop regeneration. A related example is provided by *Kalanchoe*, where epiphyllous plantlet formation depends not only on morphogenic regulators, but also on permissive chromatin states that allow differentiated leaf cells to reactivate totipotency programs (Li et al., 2011; Meng et al., 2026). This suggests that comparative chromatin analyses in Gesneriaceae and Podostemaceae may be important for understanding organogenesis at non-canonical sites.

A second desirable phenotype is perennial or root-based regrowth. Engineering annual crops with stable post-harvest regrowth could reduce soil disturbance, improve carbon retention, limit erosion, and lower the costs of repeated sowing (Zhang et al., 2011; Olsson et al., 2024). This goal is already pursued through perennial grain breeding, *de novo* domestication, interspecific hybridization, and phenotypic selection, including perennial barley using *Hordeum bulbosum* (DeHaan and Van Tassel, 2014; Chapman et al., 2022). However, the molecular drivers required to stabilize perennial traits in high-yielding backgrounds remain incompletely understood (Zhao and Wang, 2024). Podostemaceae show that root-derived tissues can acquire shoot-forming competence under specific developmental conditions. *WOX/WUS*-, *STM*-, and *ARP*-associated modules are therefore plausible candidates for testing, although their transferability to crops remains unknown. Relevant targets include cereals and oilseed crops in which controlled regrowth could support low-input or regenerative agriculture.

A third desirable phenotype is compact high-biomass architecture. This trait is relevant for controlled-environment agriculture, vertical farming, high-density cultivation, forage production, and biomass crops, where productivity depends not only on yield per plant but also on yield per unit volume, time, and input. *Lemnaceae* offer an extreme natural example of architectural compression associated with rapid clonal multiplication and reduced organ complexity. These features make them useful comparative systems for identifying regulatory configurations that support fast vegetative growth, reduced internode elongation, and efficient surface-area deployment. More broadly, controlled modulation of shoot meristem dynamics and internode elongation could provide a form of biological compression, increasing foliar density and volumetric productivity in high-density or controlled-environment cultivation systems (Teo and Yu, 2024). Such strategies remain speculative and will require functional validation in crop species. If precisely tuned, however, they may help separate vegetative compactness from reduced reproductive performance, thereby avoiding some of the pleiotropic effects frequently associated with traditional hormone-based dwarfing genes, including reduced grain size or impaired stress resilience (Li et al., 2024a, b). Candidate pathways may include *CUC*-dependent boundary and meristem programs, branching regulators, and hormone-mediated feedback or feedforward mechanisms controlling organ initiation and growth. A related example is provided by the highly conserved *CLV*/*CLE* signaling pathway, which has already been exploited during domestication and crop improvement to modulate meristem size and increase fruit, seed, or inflorescence traits in species such as tomato, maize, and apple (Xu et al., 2015; Yuste-Lisbona et al., 2020; Liu et al., 2021; Gelinas Belanger, 2025). Recent work shows that promoter editing or other quantitative modulation of *CLE* genes can dampen *CLV* signaling, producing more controlled changes in meristem activity and organ number (Lindsay et al., 2024). This supports the broader idea that crop redesign may be more effectively achieved through dosage-sensitive tuning of conserved developmental networks than through drastic loss-of-function mutations. As in polyploids, where altered gene dosage can reshape developmental outputs, this strategy offers a potentially useful but context-dependent route to crop improvement (Wang et al., 2025).

Similarly, *CUC* genes and related boundary/meristem regulators remain candidate components for testing compactness, organ initiation, and prolonged vegetative growth (Miao et al., 2024). However, their precise expression and function in Lemnaceae and other non-canonical lineages remain insufficiently characterized. Their agronomic relevance therefore depends on future validation in both the original comparative systems and in target crops.

Together, these examples illustrate how non-canonical regulatory configurations may inform regenerative biotechnology and crop architecture, provided that their functional basis and agronomic consequences are experimentally validated. Morphological diversification in *Brassica oleracea*, for example, is associated with shifts in gene expression in which transposable elements can function as dosage modulators (Cheng et al., 2016; Zhang et al., 2017; Miao et al., 2024). Such cases reinforce the idea that crop-relevant developmental innovation may arise from quantitative changes in conserved regulatory networks, from altered chromatin competence, or from the release of pre-existing developmental potential, rather than necessarily from the acquisition of entirely novel genes.

**Figure 2:**
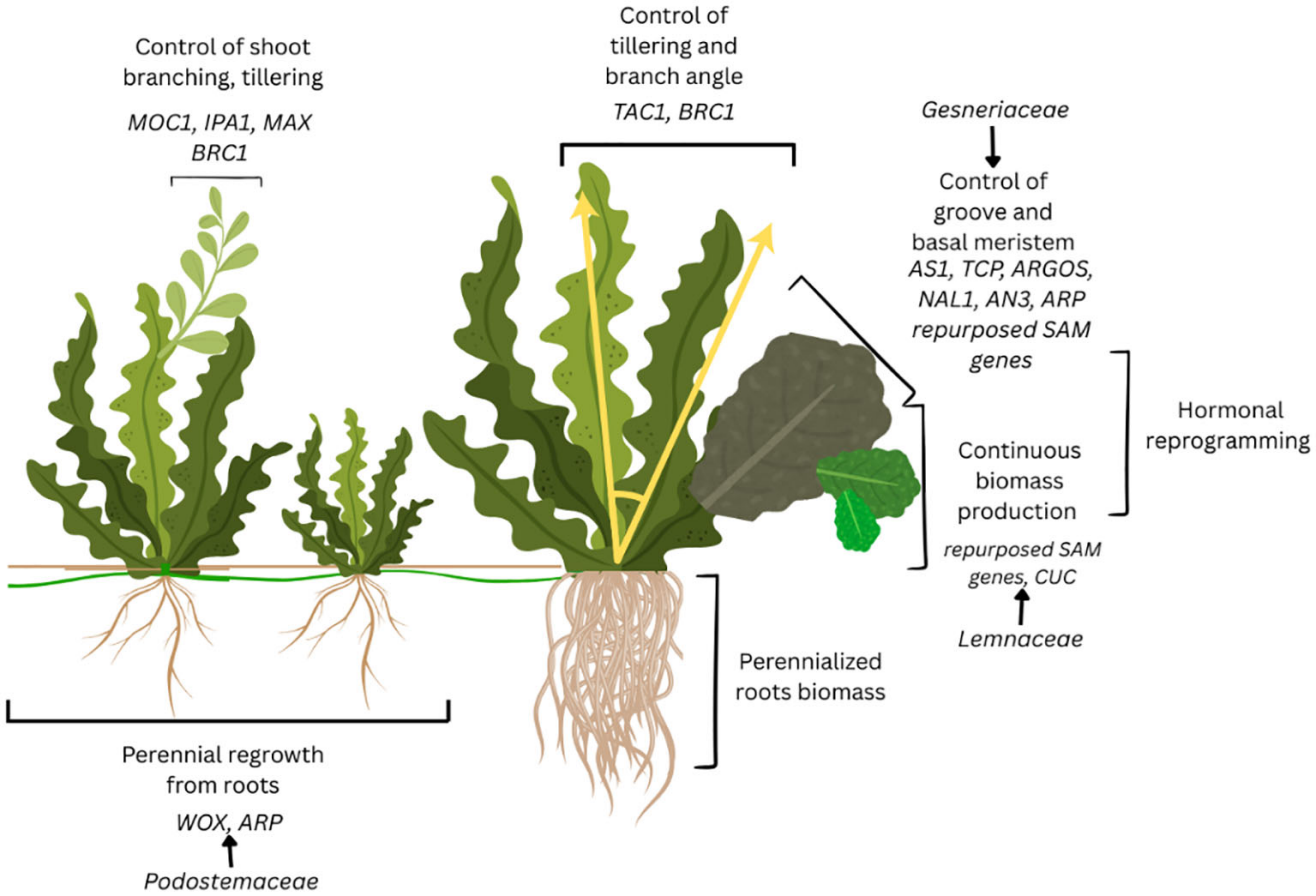
Translating non-canonical morphogenic programs into conceptual crop redesign strategies. The image illustrates how developmental innovations from non-model lineages may inform future engineering strategies for crop architecture. The genes and pathways indicated should be interpreted as candidate regulatory modules whose expression patterns and functional roles remain incompletely characterized in these lineages. (Left) Podostemaceae suggest that root-derived tissues can acquire competence for shoot organogenesis; *WOX/WUS*-, *ARP*-, and related developmental pathways represent plausible candidates for studying perennial or root-based regrowth. (Center) The fine-tuning of plant habit, including tiller number and branch angle, is achievable in crops by targeting established regulators such as *TAC1* and *BRC1*. (Right) Specialized lineages offer conceptual strategies for biomass optimization. Gesneriaceae provide examples of meristematic niches in non-canonical tissues, including groove and basal meristems, whereas Lemnaceae illustrate extreme architectural reduction associated with rapid vegetative growth. These systems do not yet constitute validated biotechnological platforms, but they provide comparative models for identifying regulatory principles that may help reshape crop ideotypes for enhanced resilience, perenniality, and productivity after functional validation.

Overall, these lineages illustrate how divergent body plans can emerge through the reconfiguration of ancient developmental circuits. In Gesneriaceae, meristem function is redistributed into leaf-like organs; in Podostemaceae, shoot identity can be recruited within root-derived tissues; and in Lemnaceae, architecture is strongly compressed. These systems suggest that the SAM is one realization of a flexible regulatory network (Zhang et al., 2021) rather than an absolute architectural requirement. Future progress will require spatial expression analyses, functional genetics, chromatin profiling, transformation systems for non-model species, and validation in target crops. **Figure 2** summarizes this framework, linking non-canonical plant architectures to candidate regulatory modules and potential agronomic phenotypes.
